# Driving forces of the humification process in composting: from a microbial perspective

**DOI:** 10.1128/aem.00207-26

**Published:** 2026-03-13

**Authors:** Jun Wang, Yaoning Chen, Hui Li, Yuanping Li, Hongjuan Jiang, Kunhong Jiang, Nan Wang, Yihang He, Zhigang Yi, Xing Peng

**Affiliations:** 1 College of Environmental Science and Engineering, Hunan University and Key Laboratory of Environmental Biology and Pollution Control (Hunan University), Ministry of Education 629143 , Changsha, China; 2 State Key Laboratory of Utilization of Woody Oil Resource, Hunan Academy of Forestry 373940 , Changsha, China; 3 School of Municipal and Geomatics Engineering, Hunan City University 66469, Yiyang, China; 4 Hunan Renhe Environmental Technology Co., Ltd., Changsha, China; University of Nebraska-Lincoln, Lincoln, Nebraska, USA

**Keywords:** composting, humification process, extracellular and intracellular metabolism

## Abstract

In composting, humification is critical for final compost quality and effectiveness and involves small molecules polymerizing into humic substances (HS). Microbial metabolism, as the core process of composting, drives this transformation. However, the full relationship between microbial metabolic processes and humification-related polymerization remains unclear in current research. This review first examines humification polymerization mechanisms from the perspective of precursors through functional group reactions and radical oxidative coupling. Then, it systematically elucidates the central role of microbial extracellular and intracellular metabolisms in driving the humification process. On the one hand, the effects of hydrolases and oxidoreductases of extracellular metabolism on the polymerization are elaborated in detail. On the other hand, it delves into the dual contributions of intracellular metabolism: catabolism supplies essential precursors and active energy for polymerization reactions through substance degradation and oxidative phosphorylation, while anabolism is directly involved in the biosynthesis of humus precursors. This work aims to provide a comprehensive theoretical framework for understanding the microbial driving mechanisms behind humification.

## INTRODUCTION

In recent years, the increasing production of organic wastes has gained significant attention in the process of agricultural and livestock production (1, 2). These organic wastes, including livestock manure, sludge, and straw, contain substantial amounts of recyclable substances such as proteins and lignocellulose. The proper disposal and recycling of these organic wastes have become an urgent priority (3), as improper management can lead to soil pollution and water eutrophication (4, 5). Some technologies, including anaerobic digestion, composting, pyrolysis, etc., have been identified in previous studies as viable options for recycling organic waste (6, 7). Composting is a cost-effective, non-hazardous, and practical large-scale technology and could yield products suitable for soil fertilization or remediation (8, 9).

The application of compost is very dependent on its quality, which is significantly influenced by the presence of HS (10). The transformation of organic material into HS is called the humification process. It can be concisely summarized as follows: the degradation of macromolecular materials into smaller molecules known as HS precursors, followed by a substantial polymerization reaction leading to the formation of HS (11). The generation and transformation of precursors serve as the foundation for polymerization reactions, while polymerization reactions constitute the core pathway for HS formation. At present, many studies have proposed various approaches to enhance the humification process, such as material pretreatment, exogenous additives, electric field assistance, aeration regulation, and the combination with biodrying (12–16), but these studies remain insufficient in elucidating the internal mechanisms and key driving factors underlying humification. To address this gap, this review investigates the interplay between microbial metabolism and humification.

Composting is a quasi-natural system where progress depends on microbial metabolic activities, which are closely related to the generation of humic precursors (17, 18). Under favorable conditions, microbial metabolism not only drives molecular transformations but may also induce polymerization reactions, providing valuable insights into the relationship between microbial metabolism and the humification process (19, 20). Environmental and substance factors are the two most critical elements in humification (21). The substance factor mainly refers to the production and conversion of precursors, which provides the material foundation for the polymerization reaction. The environmental factor mainly includes changes in pH and temperature, influencing the progression of the polymerization reaction (22). As composting is a biochemical process, both environmental and substance factors are ultimately induced by microbial metabolic functions in composting (23). Meanwhile, existing studies have clearly demonstrated that microbial metabolism plays an active role in the formation of HS in composting (24–26). Thus, the review deems that microbial metabolism is the main force in humification. However, the specific mechanisms underlying metabolic pathways and their contributions to the humification process remain poorly understood.

Aiming at the above knowledge gap, this review focuses on the composting process, elaborates on metabolic processes, and examines how metabolic processes influence polymerization reactions, further highlighting their role as a driving factor in humification. It offers a novel insight into understanding humification from a microbial perspective and offers a comprehensive elucidation of the specific mechanisms.

## POLYMERIZATION THEORY IN THE HUMIFICATION PROCESS

In composting, the humification process is intricately linked to the biological and biochemical processes, which can also be defined as the polymerization of small molecular compounds generated from the substance degradation by microorganisms to form HS (27).

**Fig 1:**
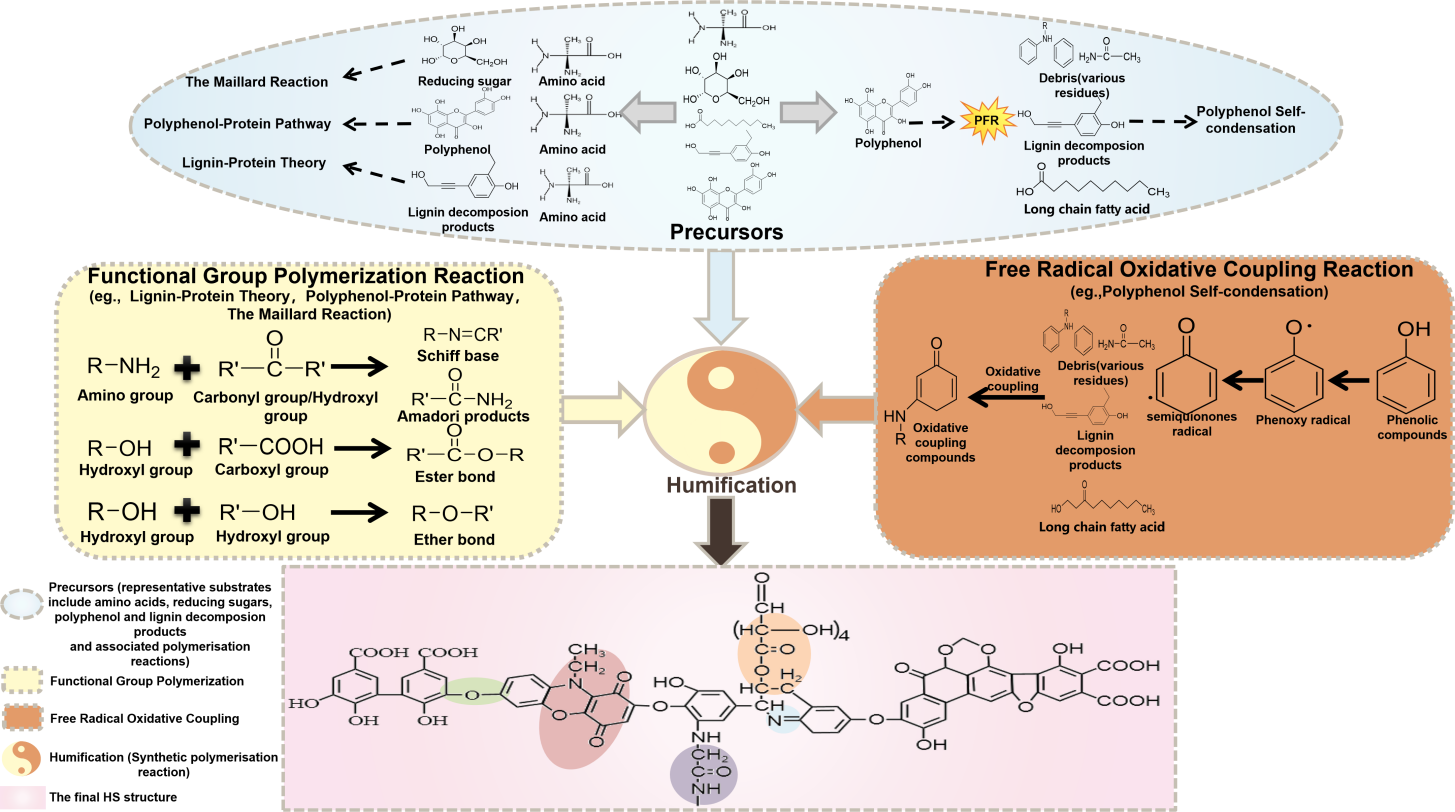
Relevant polymerization reactions in the humification process. This diagram outlines the key polymerization reactions involved in humification, integrating the four prevailing theories: (i) the lignin-protein theory, (ii) polyphenol self-condensation, (iii) the polyphenol-protein pathway, and (iv) the Maillard reaction. The process is presented in three sections: precursors (top), reaction mechanisms (center), and a structural model of the resulting humic substances (HS) (bottom). Precursors (top): Representative substrates derived from the degradation of organic matter include amino acids, reducing sugars, lignin decomposition products, long-chain fatty acids, particulate debris (various residues), and polyphenols. Reaction mechanisms (center): the left panel, focusing on functional group polymerization, illustrates the lignin-protein theory, polyphenol-protein pathway, and Maillard reaction using representative general formulas. The right panel, depicting free radical oxidative coupling, shows how phenolic compounds are transformed into PFRs, such as phenoxyl and semiquinone radicals, through electron transfer reactions. These unstable radicals subsequently undergo oxidative coupling with various precursors or metabolic byproducts to form the core skeleton of HS. Final HS structure (bottom): the proposed structure demonstrates the chemical feasibility of combining these pathways. Components are color-coded to indicate their biological or reaction origin: blue for Schiff bases, green for ether bonds, gray for Amadori products, pink for oxidative coupling compounds, and orange for ester bonds.

To date, numerous studies agree that there are four humification theories for HS formation based on different molecular structures and functional groups in humus precursors. They are the lignin-protein theory, polyphenol self-condensation, polyphenol-protein pathway, and the Maillard reaction (6, 20, 28). These theories involve two types of reactions: functional group polymerization reaction and free radical oxidative coupling (29) (Fig. 1). The first one encompasses the formation of Amadori products and Schiff bases, such as imines (C=N) and oximes (O–NH_2_), through the reaction of amino (–NH_2_) and carbonyl groups (–C=O) (9, 30). This reaction leads to the production of byproducts containing dicarbonyl compounds, such as glyoxal, methylglyoxal, and diacetyl. Additionally, as the reaction progresses, heterocyclic rings like pyrazine (C_4_N_2_H_4_) or furan (C_4_H_4_O) may be formed. Meanwhile, these reactions involving oxygen-containing compounds were intensive, such as the interaction between the hydroxyl (C–OH) and carboxyl groups (–COOH), which can form ether (C–O–C) and ester (–COOC) compounds (31, 32). These newly formed products have active groups and a higher electron cloud density of heterocyclic atoms (N, O) within the heterocyclic ring; thus, they will undergo continuous binding with proteins and lignin residues by group reaction, forming hydrogen bonds and stacking of π-π electrons (33, 34). Consequently, these interactions lead to the formation of low molecular weight polymers, facilitating gradual polymerization. The other polymerization involving free radical oxidative coupling predominantly occurs during the conversion of phenolic compounds, such as the transformation of phenolic compounds into persistent free radicals (PFRs), such as phenoxyl radicals and semiquinone radicals. Subsequently, unstable electrons in PFRs transfer to electron acceptors with higher redox potential. Electron-losing PFRs readily undergo coupling reactions with nitrogenous or other substrates to form the skeleton of HS (35, 36).

The primary condition for the above polymerization reaction is the availability of sufficient precursors with active functional groups (16, 32). Additionally, as the humification process progresses, environmental factors, such as temperature and pH, can influence the polymerization from the rate and intensity of the reaction (37). Thus, the generation, transformation, and polymerization of precursors are not only critical drivers of humification but also key elements in elucidating the intrinsic relationship between microbial metabolism and humification. But, the specific roles of metabolic processes in this process remain incompletely understood. Therefore, subsequent contents are based on the polymerization reaction mechanism, focusing on elucidating the specific pathways through which microbial metabolism regulates the humification process.

## RELATIONSHIP BETWEEN MICROBIAL METABOLISM AND THE HUMIFICATION PROCESS

With the progress of composting, the metabolism of microorganisms will use diverse enzymes to obtain available substances and simultaneously transform these substances, and then influence other abiotic transformations of substances by changing the microenvironment (38, 39).

The metabolic activities of microorganisms in composting involve a large number of specific metabolic processes; hence, which metabolic processes and how to participate in humification need to be systematically and deeply explored (40). For example, some macromolecular substances commonly found in compost materials, such as lignocellulose, lipids, and proteins, can be degraded into primary metabolites (e.g., sugars, fatty acids, amino acids, aromatic compounds, etc.) via microbial metabolism (41). Furthermore, these substances undergo further transformation into secondary metabolites, which often contain aromatic compounds and carbonyl, carboxyl, hydroxyl, amino, and amide groups (31, 33). These two kinds of metabolites could be used as precursors to participate in polymerization to form HS through functional group polymerization and free radical oxidative coupling (28, 29). Generally, according to the location where metabolic processes occur, the overall microbial metabolism covering material and energy metabolism is discussed in this study from two main components: intracellular and extracellular metabolisms (22).

### Role of extracellular metabolism in the humification process

Extracellular metabolism is mostly involved in the biodegradation of macromolecular compounds through the action of extracellular enzymes (39, 42). These diverse enzymes can break down complex organic compounds, such as lignocellulose, starch, and proteins, into small molecules and other residues through oxidative reactions, hydrolysis, and decarboxylation (43). The generated small molecules include long-chain fatty acids (derived from lignocellulosic residues like suberin and cutin), amino acids, reducing sugars, monosaccharides, and aromatic compounds, which possess active functional groups (e.g., amino, carbonyl, and aromatic groups) capable of directly participating in functional group polymerization reactions (e.g., carbonyl-amino condensation, oxygen-containing functional group condensation, and electron cloud stacking) (9, 31). Additionally, substances with reductive functional groups undergo further transformation through extracellular metabolism, generating reactive intermediates and triggering oxidative coupling polymerization reactions (44). Hence, extracellular substance metabolisms can participate directly in humification by inducing substance transformation.

The extracellular enzymes involved in extracellular metabolism can be divided into two categories: hydrolases and oxidoreductases (45, 46). The hydrolases typically cleave specific chemical bonds through hydrolysis reactions within substrates, leading to the conversion of complex compounds into simpler ones. Studies have confirmed that the activity of hydrolases and changes in their related functional genes are significantly correlated with the formation of HS in the final compost products (47).

**Fig 2:**
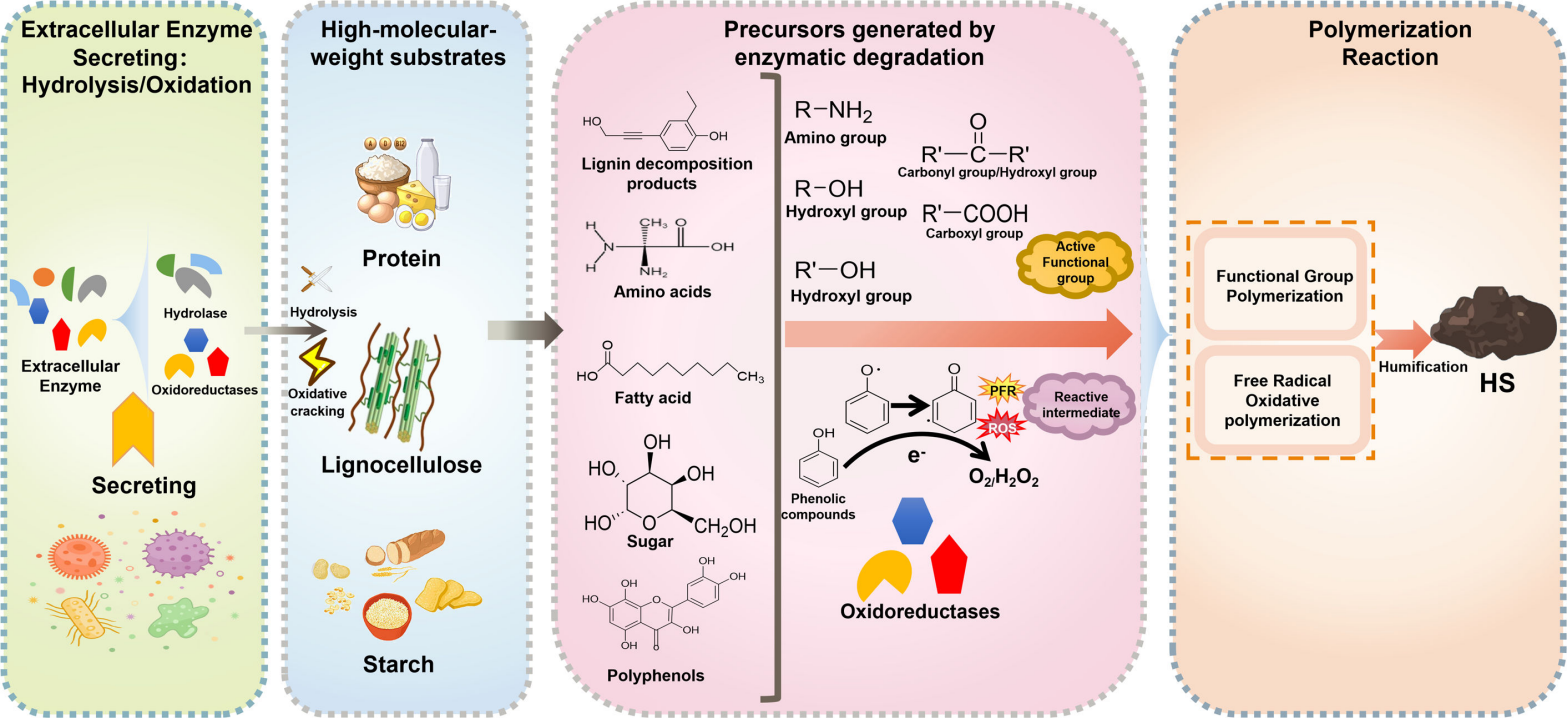
Role of extracellular metabolism in the humification process. (Pink panel) Generation of precursors: the pink panel illustrates how enzymes, including hydrolases and oxidoreductases, serve as “precursor providers.” They catalyze the hydrolysis and oxidative cleavage of bonds in macromolecules, releasing essential building blocks, such as monosaccharides, amino acids, fatty acids, polyphenols, and lignin decomposition products. These compounds supply the active functional groups necessary for initial polymerization. Furthermore, oxidoreductases directly drive the subsequent polymerization stage via electron-transfer mechanisms. By utilizing various electron acceptors, they generate reactive oxygen species (ROS) and persistent free radicals (PFRs), including phenoxyl and semiquinone radicals. These highly reactive intermediates initiate radical oxidative coupling and sustain the phenol-quinone redox cycle. This cycle facilitates cross-linking among diverse precursors, thereby enhancing the aromaticity and molecular weight of the resulting humic substances (HS).

On the contrary, oxidoreductases promote the degradation of recalcitrant substances and further drive transformations of products, operating through mechanisms that involve the generation of reactive intermediates, such as PFRs and reactive oxygen species (ROS) (7, 48). This capability allows oxidoreductases to degrade materials more efficiently, releasing aromatic compounds from lignocellulose and influencing the subsequent transformation of phenolic components. As a result, they provide more polymerization sites for HS formation. Previous studies have also indicated that the activities of relevant oxidoreductases and upregulation of their regulatory genes not only supply the primary skeleton for HS but also shape their aromaticity and unsaturation (49, 50). The types of hydrolases and oxidoreductases are summarized in Table 1. The specific mechanism of extracellular enzymes in different polymerization reactions will be discussed in detail below (Fig. 2).

**TABLE 1:** Enzymes involved in the extracellular metabolism process

Category	Enzyme	Substrate	Microbial source (representative genera and species)
Hydrolases	Endoglucanase	Cellulose	*Trichoderma* (*Trichoderma reesei*) *Penicillium* (*Penicillium verruculosum*) *Pseudomonas* (*Pseudomonas putida*) *Cellulomonas* (*Cellulomonas fimi*)
	Exoglucanase	Cellulose	*Thermothelomyces* (*Thermothelomyces thermophilus*) *Talaromyces* (*Talaromyces thermophilus*) *Thermobifida* (*Thermobifida fusca*) *Clostridium* (*Clostridium thermocellum*)
	β-Glucosidase	Cellulose	*Penicillium* (*Penicillium brasilianum*) *Neocallimastix* (*Neocallimastix patriciarum W5*) *Paenibacillus* (*Paenibacillus lautus BHU3*) *Streptomyces* (*Streptomyces* sp*. QM-B814*)
	Endoxylanase	Hemicellulose	*Thermoascus* (*Thermoascus aurantiacus*) *Aspergillus* (*Aspergillus terreus S9*) *Bacillus* (*Bacillus halodurans*) *Thermoactinomyces* (*Thermoactinomyces thalophilus*)
	Xylosidase	Hemicellulose	*Myceliophthora* (*Myceliophthora thermophila M77*) *Penicillium* (*Penicillium janczewskii*) *Meiothermus* (*Meiothermus taiwanensis WR-220*) *Bacillus* (*Bacillus pumilus*)
	Mannanase	Hemicellulose	*Aspergillus* (*Aspergillus nidulans*) *Podospora* (*Podospora anserina*) *Alicyclobacillus* (*Alicyclobacillus acidocaldarius*) *Cellvibrio* (*Cellvibrio japonicus*)
	Mannosidase	Hemicellulose	*Talaromyces* (*Talaromyces cellulolyticus*) *Pichia* (*Pichia pastoris*) *Streptomyces* (*Streptomyces* sp*. S27*) *Cellvibrio* (*Cellvibrio mixtus*)
	Endogalactanase	Hemicellulose	*Aspergillus* (*Aspergillus aculeatus*) *Bacteroides* (*Bacteroides thetaiotaomicron*) *Bifidobacterium* (*Bifidobacterium longum*)
	Galactosidase	Hemicellulose	*Rhizomucor* (*Rhizomucor miehei*) *Aspergillus* (*Aspergillus oryzae*) *Clostridium* (*Clostridium josui*) *Pseudomonas* (*Pseudomonas fluorescens*)
	Protease	Proteins	*Aspergillus* (*Aspergillus fumigatus*) *Chryseobacterium* (*Chryseobacterium aquaticum PUPC1*) *Cryptococcus* (*Cryptococcus aureus*) *Alcaligenes* (*Alcaligenes faecalis*)
	Amylase	Starch	*Aspergillus* (*Aspergillus niger*) *Coprinus* (*Coprinus comatus*) *Lactobacillus* (*Lactobacillus* sp. *RKY2*) *Thalassobacillus* (*Thalassobacillus* sp*. LY18*)
	Lipase	Lipids	*Candida* (*Candida antarctica*) *Aspergillus* (*Aspergillus awamori BTMFW032*) *Pseudomonas* (*Pseudomonas aeruginosa*) *Lactobacillus* (*Lactobacillus plantarum*)
	Chitinase	Chitin	*Trichoderma* (*Trichoderma viride*) *Nosema* (*Nosema bombycis*) *Stenotrophomonas* (*Stenotrophomonas maltophilia*) *Serratia* (*Serratia marcescens*)
	Pectinase	Pectin	*Pycnoporus* (*Pycnoporus sanguineus*) *Rhizopus* (*Rhizopus oryzae*) *Kosakonia* (*Kosakonia sacchari TD2*) *Acinetobacter* (*Acinetobacter guillouiae TD1*)
Oxideoreductases	Laccase	Lignin	*Fusarium* (*Fusarium udum*) *Pycnoporus* (*Pycnoporus cinnabarinus*) *Streptomyces* (*Streptomyces cyaneus*) *Geobacillus* (*Geobacillus thermocatenulatus*)
	Lytic polysaccharide monooxygenase	Cellulose	*Neurospora* (*Neurospora crassa*) *Malbranchea* (*Malbranchea cinnamomea*) *Cellvibrio* (*Cellvibrio japonicus*) *Streptomyces* (*Streptomyces coelicolor*)
	Cellobiose dehydrogenase	Cellulose	*Myriococcum* (*Myriococcum thermophilum*) *Sporotrichum* (*Sporotrichum thermophile*) *Cellulomonas* (*Cellulomonas palmilytica EW123*)
	Lignin peroxidase	Lignin	*Irpex* (*Irpex lacteus*) *Panus* (*Panus coccineus*) *Streptomyces* (*Streptomyces viridosporus T7A*) *Rhodococcus* (*Rhodococcus jostii RHA1*)
	Manganese peroxidase	Lignin	*Rigidoporus* (*Rigidoporus lignosus*) *Pleurotus* (*Pleurotus ostreatus*) *Bacillus* (*Bacillus circulans*) *Pseudomonas* (*Pseudomonas aeruginosa M10*)
	DyP-type peroxidase	Lignin	*Bjerkandera* (*Bjerkandera adusta*) *Geotrichum* (*Geotrichum candidum*) *Streptomyces* (*Streptomyces avermitilis*) *Saccharomonospora* (*Saccharomonospora viridis*)

#### Effect of hydrolases on the polymerization

Hydrolases mainly play a role in providing precursors for polymerization via the hydrolysis of lignocellulose and protein in composting (51). The lignocellulose contains lignin, cellulose, and hemicellulose. Cellulose is a polysaccharide polymer composed primarily of glucose molecules linked together by *β*-1,4-glycosidic bonds, forming a linear chain structure. As a cellulose hydrolase, endoglucanase can catalyze the cleavage of *β*-1,4-glycosidic bonds in the non-crystalline region of cellulose, leading to the generation of oligosaccharides and exposing both reducing and non-reducing end groups (49). Subsequently, exoglucosidase acts upon these end groups to form the cellulose dimers, which are further hydrolyzed into glucose by the action of *β*-glucosidase (45, 46). As for the degradation of hemicellulose, the hydrolysis enzymes involve mainly endoxylanase, xylosidase, mannanase, mannosidase, endogalactanase, and galactosidase (52). Endoxylanase, xylosidase, and mannanase specifically hydrolyze xylan backbone bonds, xylan-polysaccharide linkages, and mannan chain bonds in hemicellulose, respectively. They can also be decomposed into dimers. Subsequently, dimers can be degraded into xylose, mannose, and galactose (53). Proteins are readily degradable substances that can be hydrolyzed by proteases to produce peptides and amino acids (54). These hydrolysates could be precursors comprising various monosaccharides and amino acids, which are capable of directly participating in the polymerization reaction due to their active functional groups (carbonyl and amino group) to form complex polymeric structures for humification (33).

#### Effect of oxidoreductases on the polymerization reaction

Compared with hydrolases, oxidoreductases possess unique redox capabilities that play a critical role in the generation and transformation of precursors, thereby establishing the foundation for subsequent complex polymerization reactions. This distinctive ability arises from the enzyme-mediated electron transfer mechanism (7). Notably, the redox capacity of oxidoreductases is primarily determined by the redox potential of their final electron acceptors (55). Based on this characteristic, oxidoreductases are classified into two categories based on their different final electron acceptors: oxygen- and hydrogen peroxide-utilizing (52). The enzymes using O_2_ (E^0^ = +1.3 V) as an electron acceptor, including laccase (Lac) and lytic polysaccharide monooxygenases (LPMO) (56), exhibit relatively weaker oxidizing power due to the lower redox potential and primarily target reducible functional groups, such as phenolic hydroxyl groups for oxidation (50). These enzymes are abundant in composting environments due to the availability of oxygen (15). In contrast, peroxidases, such as lignin peroxidase (LiP) and manganese peroxidase (MnP), use H_2_O_2_ (E^0^ = +1.78V) as an electron acceptor and exhibit higher redox potentials and stronger oxidizing capacities, enabling them to directly act on recalcitrant alkyl bonds (47).

Generally, oxidoreductases can degrade lignin and cellulose in direct and indirect ways, respectively (44, 45). In indirect degradation, oxidoreductases have the potential to induce the production of reactive intermediates through single electron transfer, regardless of whether oxygen or hydrogen peroxide serves as the electron acceptor. The reactive intermediates include ROS (•OH, ^•^O_2_^−^, and H_2_O_2_) and PFRs. Due to their higher redox potentials (E^0^ = +2.8 V, −0.33 V, and +1.78V for •OH, ^•^O_2_^−^, and H_2_O_2_, respectively), ROS can break the chemical bonds of macromolecular substances and release precursors, such as polyphenols, lignin residues, and polysaccharides (57, 58). In direct degradation, oxidoreductase can break chemical bonds by acquiring electrons from ether bonds, C_α_–C_β_ bonds, and alkyl bonds in lignocellulose, and then inserting oxygen atoms to produce more oxygen-containing functional groups, such as hydroxyl, carboxyl, and carbonyl groups (27, 44). These generated substances can be the precursors that undergo functional group polymerization to form HS.

In addition to functional group polymerization, radical oxidative coupling is another polymerization reaction induced by the occurrence of phenolic compounds (48). This is attributed to the further transformation of phenolic components by oxidoreductase, which can extract electrons from phenol compounds to the final electron acceptors, leading to the generation of reactive free radical intermediates, such as phenoxy and semiquinone radicals (36). The semiquinone radical is a more stable compound that can undergo oxidative coupling with other nitrogenous substances and aromatic substances. Meantime, this process also plays a significant role in the redox cycle associated with the generation of ROS (59). Sun et al. found that the generation of ROS is promoted in the phenol-quinone cycle, imparting significant oxidative capacity to the process (48). This oxidative capacity not only accelerates the further transformation of more reductive substances but also induces their sustained involvement in polymerization reactions, ultimately leading to the formation of high-molecular-weight oxidative coupling products (35). From the perspective of ROS, their high redox potential not only renders them critical in breaking chemical bonds but also serves as the primary driving force for radical oxidative coupling polymerization reactions (29). Thus, investigating the effect of the phenol-quinone cycle on compost from the perspectives of free radical production and humification represents a novel research perspective.

### Role of intracellular metabolism in the humification process

As the composting process proceeds, various small-molecule substances, also known as primary metabolites, are released from macromolecular substances (60). Microbes typically utilize these substances to support their growth and activity through intracellular metabolism (Fig. 4) (61). This metabolism involves the production of energy and secondary metabolites, which often leads to changes in the microenvironment and the composition of composting (20). Notably, polymerization reactions still often occur concurrently with these metabolic processes. Because of the difficulty of direct investigation, intracellular metabolism is often explored using molecular biology methods, including metabolomic analysis, metagenomic analysis, and computational tools for microbial functional gene prediction, such as the Phylogenetic Investigation of Communities by Reconstruction of Unobserved States (PICRUST), Tax4Fun, Functional Annotation of Prokaryotic Taxa (FAPROTAX), and BugBase (46, 62, 63).

Metagenomic analysis can generate a non-redundant gene catalog for functional gene analysis. Concurrently, phylogenetic marker gene data obtained from 16S rRNA sequencing can be used to predict functional gene abundance (25, 64). Metabolomic analysis detects metabolites using techniques, such as liquid chromatography-mass spectrometry (LC-MS) or gas chromatography-mass spectrometry (GC-MS), followed by statistical methods to explore the dynamic multiparametric metabolic response (63).

Analyses of intracellular metabolic processes facilitated by the Kyoto Encyclopedia of Genes and Genomes (KEGG) database highlight that metabolisms in carbohydrates, amino acids, xenobiotics, and lipids are crucial for humification (65). Several specific KEGG Level 3 pathways within these categories have been confirmed to influence humus formation (62). These include (i) glycolysis pathway (GP), pentose phosphate pathway (PPP), and tricarboxylic acid cycle (TCA) from carbohydrate metabolism, (ii) tryptophan metabolism and tyrosine metabolism from amino acid metabolism, (iii) bisphenol metabolism and quinone biosynthesis from xenobiotics metabolism, and (iv) fatty acid degradation from lipid metabolism (65). These pathways involve both catabolism and anabolism. However, the precise mechanisms by which they drive polymerization reactions remain incompletely understood, underscoring the need for further investigation.

#### How can catabolism influence polymerization reaction?

##### Role of substance degradation in catabolism

In previous studies, catabolism has typically been associated with energy metabolism and the generation of metabolic intermediates, a process that induces changes in environmental conditions and material composition during composting. Consequently, it is speculated that catabolism can directly or indirectly impact polymerization reactions by decomposing small-molecule substances like saccharides, amino and fatty acids (66).

In the process of catabolism, small molecular substances are converted into pyruvate and acetyl-CoA via specific metabolic pathways and subsequently enter the tricarboxylic acid (TCA) cycle, ultimately yielding adenosine triphosphate (ATP) and reduced nicotinamide adenine dinucleotide (NADH) (67). Notably, the TCA cycle serves as a central pathway for energy metabolism and is often accompanied by the generation of various byproducts while serving as the primary driver of temperature increases and carbon dioxide emissions during composting (25, 68).

The transformation of saccharides usually involves the GP and PPP, which undergo decarboxylation and redox reactions through relevant key enzymes, leading to the formation of ketones, short-chain carboxylic acids, and alcohols (69, 70). The amino acid metabolism has various degradation pathways, such as alanine, threonine, lysine, aspartate, and glutamate, which can undergo deamination and decarboxylation reactions, resulting in intermediates, such as organic acids and amides (38, 71). Similarly, fatty acids can be converted into glycerol and short-chain fatty acids through decarboxylation (15). These intermediate products possess active functional groups, such as hydroxyl, carbonyl, and carboxylic acid groups, which endow them with the potential to participate in functional group polymerization reactions. Meanwhile, amides identified as the typical metabolic intermediate are active substances that can provide additional binding sites to enhance polymerization processes and increase the molecular weight of polymers (38).

Overall, these metabolic processes produce intermediates through a series of deoxygenation, decarboxylation, ketogenesis, and esterification reactions, and these intermediates with active functional groups can significantly enhance the process of polymerization for humification.

Additionally, changes in temperature and pH induced by microbial metabolism could increase the potential for polymerization reactions between substances. As a core pathway of energy metabolism, the fundamental energy generation of catabolism lies in the redox reactions of chemical compounds. Within microorganisms, this process involves multiple enzymatic steps and a series of intermediates, through which redox-active compounds are transferred along metabolic pathways. This process is accompanied by a significant thermal impact, thereby linking catabolism closely to changes in system temperature (72). In particular, the TCA cycle, as a central metabolic pathway, plays a key role in raising the temperature within composting systems, and the heat it releases provides the necessary activation energy for subsequent polymerization reactions (32, 73). Numerous studies have confirmed that suitable thermal conditions are critical for the functional group polymerization reaction and the free radical oxidative coupling (74, 75). Research indicates that ketonization and hydroxylation reactions tend to proceed readily in the system under suitable temperature, promoting intramolecular functional group transformation and increasing the content of oxygen-containing functional groups, such as carbonyl and hydroxyl groups. These changes further drive polymerization reactions, such as amine-aldehyde condensation or hydroxylamine condensation. Meanwhile, thermally labile compounds can undergo oxidative dehydrogenation at active sites, generating unstable radical intermediates. These intermediates with aromatic structures subsequently undergo spontaneous polymerization through radical coupling and hydrogen bonding, ultimately forming highly cross-linked polyphenolic self-dimers or co-dimers (31, 74). This series of thermally driven reaction processes is also one of the important reasons why thermophilic composting can efficiently achieve rapid humification. As for pH, the deamination reaction releases amino groups into the environment, which can lead to a pH increase in the composting (76). The alkaline conditions are conducive to polymerization reactions in the humification (37). In the presence of alkali and oxygen, carbohydrates and organic acids will undergo decarboxylation, leading to the generation of aromatic hydrocarbons (31). These aromatic hydrocarbons subsequently undergo structural crosslinking with oxygen-rich polymerization intermediates, thereby enhancing the molecular weight of the polymer (77). This process also further contributes to the formation of supramolecular humic-like structures.

##### Role of the oxidative phosphorylation process in catabolism

Oxidative phosphorylation, as a key process in energy metabolism, is a central electron transfer pathway in catabolism (78). During this process, metabolic substrates release electrons, which are accepted by NAD^+^ and FAD^+^ to form NADH and FADH_2_, respectively. These reduced coenzymes enter the electron transport chain, transferring electrons to the terminal electron acceptor. Concurrently, the generation of a proton drives ATP synthase to synthesize ATP from ADP and inorganic phosphate (Pi) (79).

**Fig 3:**
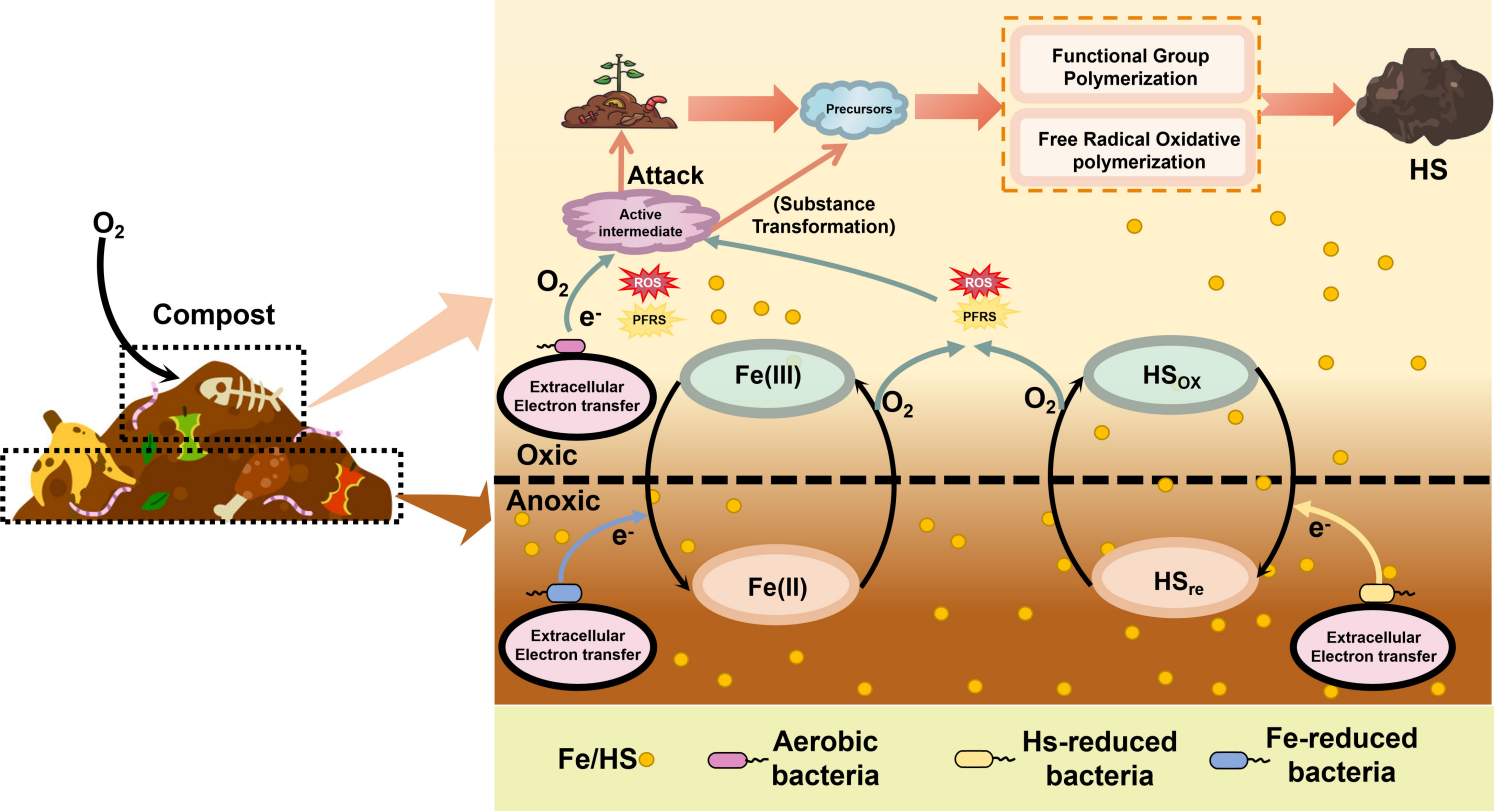
Mechanism of EET driving the humification process in composting. The schematic illustrates the pathway of electron translocation from intracellular oxidative phosphorylation to extracellular electron acceptors like Fe(III) and humic substances in different redox states (HSox/HSre) within the compost matrix. Metabolic substrates release electrons via the electron transport chain, facilitating the reduction of extracellular acceptors. This interaction induces the generation of key reactive intermediates: reactive oxygen species (ROS) and persistent free radicals (PFRs). These intermediates regulate the extracellular redox gradient and accelerate the oxidation of organic precursors, thereby promoting polymerization reactions that lead to the formation and stabilization of humic substances (HS).

Moreover, the electron transfer process can be divided into extracellular and intracellular processes depending on whether the final electron receptor is inside or outside the cell (80). Extracellular electron transfer (EET) involves the translocation of electrons across the cellular membrane to extracellular electron acceptors, such as oxygen, iron, or HS (Fig. 3) (81, 82). It can induce redox state variations in the extracellular environment, thereby driving organic matter oxidation and carbon turnover processes (83). From a mechanistic perspective, the extracellular receptors can be reduced by direct or indirect interaction with the electron transport chain after receiving electrons. This reduction induces the generation of key reactive intermediates, ROS, and PFRs, which play a critical role in facilitating the transformation and generation of precursors (59, 84, 85). The production of ROS is closely associated with key enzymes in the electron transfer chain, such as NADH oxidoreductases and cytochrome c oxidoreductases (86, 87). Based on the above research evidence, this review speculates EET may regulate the generation and transformation of extracellular precursors by modulating extracellular redox gradients, contributing to the promotion of polymerization reactions.

Despite the lack of conclusive evidence establishing a direct effect of EET on the humification process during composting, the mixed aerobic-anaerobic conditions inherent to composting systems suggest that EET processes should objectively occur (88). Within this context, potential relationships between EET-functional microorganisms and humification have been examined. It has been confirmed that the addition of iron minerals in composting cannot only establish Fenton or Fenton-like reactions but also benefit the enrichment of iron-reducing bacteria (EET function), such as *Bacillus*, *Geobacillus*, *Ferribacterium*, *Rhodobacter*, *Paenibacillus*, *Brevibacillus*, and *Ferruginibacter*, driving the iron cycle and inducing the generation of ROS and PFRs. It significantly enhanced the oxidation rate of organic matter and carbon conversion efficiency, thereby effectively promoting the humification process (59, 89). Similarly, electric field-assisted composting can also enhance electron transfer efficiency, significantly increasing the abundance of electroactive bacteria, such as *Bacillus*, *Enterococcus*, and *Tepidimicrobium*. Thus, EET-functional microorganisms’ enhancement further promotes the formation of reactive intermediates, exerting a significant driving force on the humification process (14). Notably, as the humification process progresses, the production of HS not only facilitates the enrichment of humus-reducing microorganisms but also promotes the further polymerization of aromatic group- and oxygen-containing functional group-rich substances. This process enhances the final HS aromaticity and unsaturation, thereby strengthening their electron transfer capacity and application potential (90).

In conclusion, the enrichment of EET-capable microorganisms could enhance redox reactions during composting, significantly improving the efficiency of organic matter conversion and exerting a pronounced promoting effect on the humification process.

### How can anabolism influence polymerization reaction?

**Fig 4:**
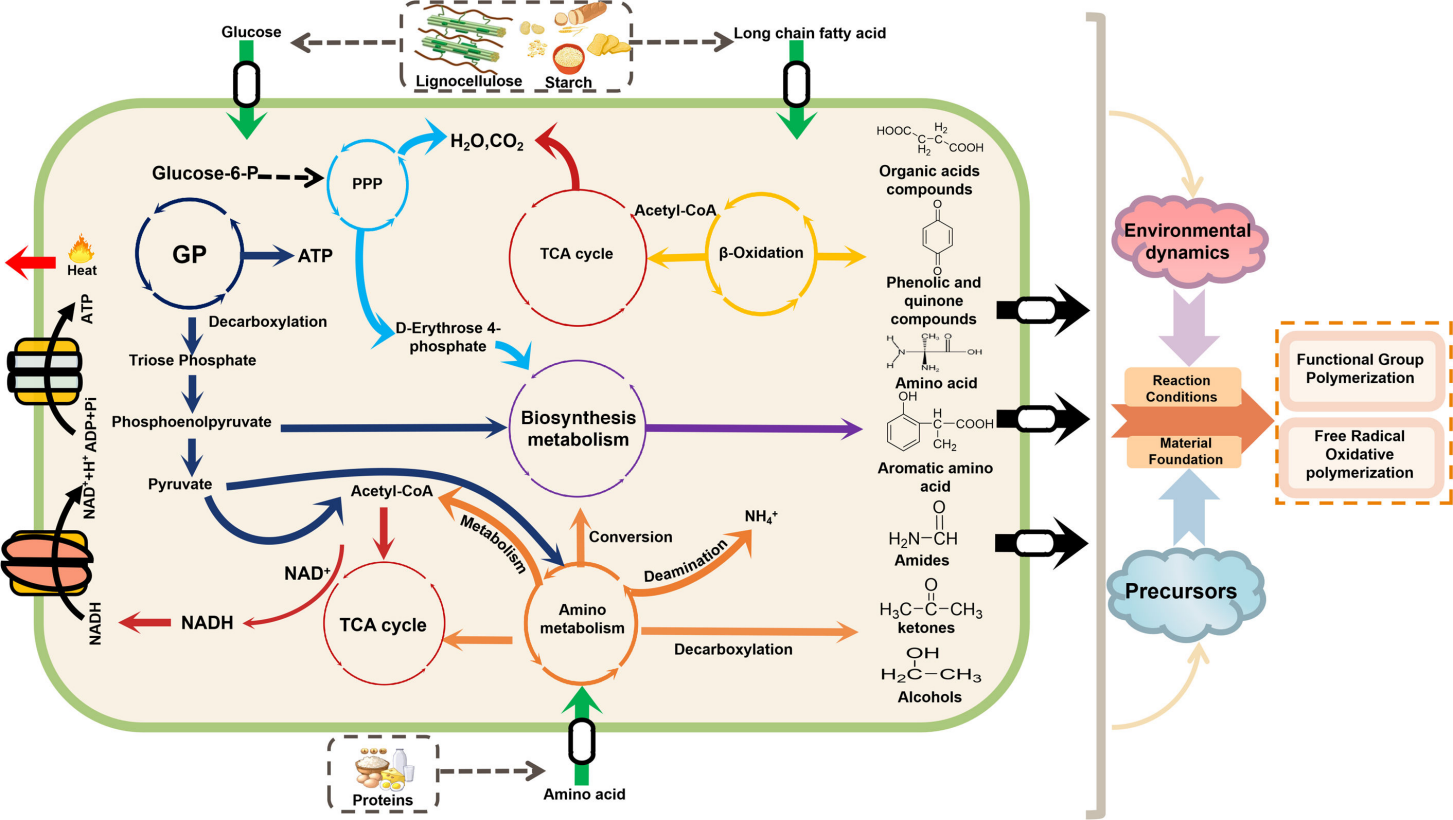
Intracellular metabolic mechanisms in humification polymerization. This diagram illustrates how microbial intracellular metabolism provides both the material foundation and the reaction conditions essential for humus formation. Catabolism utilizes small-molecule carbon sources to generate energy (ATP), heat, and intermediate metabolites. These intermediates are then channeled into anabolic biosynthesis pathways. The resulting biosynthetic precursors are subsequently transported out of the cell. In the extracellular environment, they undergo functional group polymerization and free-radical oxidative coupling to form humic substances (HS). This extracellular polymerization is further promoted by catabolically generated environmental dynamics, such as bioheat and pH changes. (GP: glycolysis pathway; PPP: pentose phosphate pathway; TCA cycle: tricarboxylic acid cycle; acetyl-CoA: acetyl coenzyme A; NADH/NAD+: nicotinamide adenine dinucleotide; ATP/ADP: adenosine triphosphate/diphosphate).

As a critical link in integrating intermediate metabolites generated during catabolism, anabolism plays a significant role in the humification process by converting these intermediates into biomolecules, thereby providing the material foundation for subsequent polymerization reactions (Fig. 4). The biosynthesis of amino acids and aromatic compounds, including amino and aromatic amino acids, phenol, and quinone compounds, is of particular importance because these compounds often serve as precursors in polymerization reactions during humification (91, 92). According to the KEGG database, amino acid metabolism includes amino acid degradation and biosynthesis (46). Specifically, the biosynthesis of amino acids can be categorized into aromatic amino acids and non-aromatic amino acids, such as glycine, glutamic acid, serine, threonine, valine, leucine, and isoleucine (11, 62). These biosynthetic metabolites are significantly positively correlated with the accumulation of HS, although the specific mechanisms and direct relationship between them remain to be further investigated (65).

The ammonia assimilation, characterized by a well-defined metabolic pathway and nitrogen fixation mechanism, has become a representative pathway for studying the relationship between amino acid synthesis metabolism and humification in composting research (93). This pathway facilitates the conversion of NH^4+^-N to organic-N (glutamine and glutamic acid) via the GDH and GS-GOGAT pathway, with key enzymes, including glutamate dehydrogenase (GDH) and glutamine synthetase (GOGAT) (43). In bio-enhanced mechanical composting (BMC), which employs mechanical turning, forced aeration, and external heating to promote evaporation and bioheat production, the ammonia assimilation capacity is significantly improved by the accumulation of organic acids and the consequent pH decrease (76). Consequently, within the BMC system, the activities of key enzymes involved in the GDH and GS-GOGAT pathways are upregulated and accompanied by increased expression of related regulatory genes (*gdh*, *glnA*, and *amtB*). These changes collectively enhance amino acid biosynthesis and ultimately promote the formation of humus. Supporting this mechanism, Liang et al. further confirmed that microbial inoculation enhances the ammonia assimilation pathway and upregulates related genes, which are positively correlated with the degree of compost humification (94). These findings not only clarify the theoretical link between amino acid biosynthetic pathways and humification but also provide direct experimental evidence supporting this connection.

Aromatic amino acids, such as phenylalanine, tyrosine, and tryptophan, possess aromatic rings and diverse functional groups, enabling them to induce more complex polymerization reactions (70). The shikimic acid pathway (SAP), a key metabolic pathway for aromatic amino acid biosynthesis, is widely utilized in composting studies to elucidate the intrinsic connections between microbial metabolism and humification. This pathway uses intermediate metabolites produced during catabolism, such as erythrose-4-phosphate (E4P) and phosphoenolpyruvate (PEP), as precursors to synthesize aromatic amino acids through a series of enzymatic reactions (95). Key enzymes include 3-deoxy-7-phosphoheptaketose isomerase (encoded by the *aroB* gene), dehydroquinate dehydratase (encoded by the *Quic* gene), and shikimate dehydrogenase (encoded by the *SDH* gene) (47). The expression levels of these key enzyme-encoding genes are significantly upregulated under various regulatory strategies that promote humification (20). This phenomenon is associated with microbial strategies for mitigating oxidative damage caused by extracellular redox fluctuations. Tang et al. have shown that when extracellular redox fluctuations trigger microbial oxidative stress, microorganisms typically upregulate the PPP, generating important metabolic intermediates such as PEP and E4P. These intermediates further enter the SAP, ultimately leading to the synthesis of aromatic compounds, thereby helping microorganisms alleviate oxidative damage and maintain intracellular redox balance (70). Similarly, studies employing oxidative strategies to promote humification during composting, such as hyperthermophilic pretreatment and in-situ Fenton reactions, have consistently observed a significant upregulation of genes associated with the SAP, further confirming its crucial role in the humification process (70, 96). This discovery highlights the pivotal role of the SAP in humification and provides critical theoretical insights for optimizing composting through microbial metabolic regulation.

In addition to aromatic amino acids, a major branch of the SAP can synthesize intracellular phenolic aromatic compounds, such as catechol and gallic acid (22, 91). These phenolic compounds directly participate in functional group polymerization reactions and facilitate oxidative coupling polymerization through redox cycles involving the conversion of phenol-quinone (48). The strong correlation between the biosynthetic pathways of phenols and quinones and the humification process has been revealed by the KEGG database and NIST standards in composting (63). These findings underscore the direct impact of aromatic compound biosynthesis during anabolism on the humification process.

To sum up, on the one hand, catabolism involves the decomposition of carbon sources into intermediate metabolites with active functional groups and binding sites, which serve as key precursors for extracellular polymerization reactions. This process not only significantly alters the physicochemical properties of the extracellular environment (e.g., pH) but also releases energy and heat, providing the activation energy required for extracellular polymerization reactions. Furthermore, oxidative phosphorylation during catabolism drives extracellular redox reactions, enabling chemical transformations of precursor substances and facilitating more complex polymerization reactions. On the other hand, anabolism integrates a portion of the intermediates generated during catabolism to synthesize macromolecules or functional molecules, thereby providing the essential material foundation for the humification process.

## CONCLUSION

This review systematically investigates the interaction mechanisms between microbial metabolisms and the humification process in composting systems, highlighting the pivotal role of microorganisms in driving the humification process. It is found that microorganisms decompose large organic molecules into small precursor substances through metabolic activities, which further form stable humus structures via functional group polymerization reactions and radical linkages. Specifically, extracellular metabolism secretes extracellular enzymes to decompose organic matter, generating and transforming key precursors, thereby providing the material foundation for humification. Intracellular metabolism produces metabolites that not only provide the necessary material support for polymerization reactions but also regulate the extracellular environmental conditions to create favorable reaction conditions for the humification process. Although these findings provide significant references for the theoretical construction of humification mechanisms and the optimization of composting processes, there is still a notable knowledge gap in understanding the direct interactions between specific metabolic pathways and the humification process. Future work should prioritize the integration of molecular biology techniques, such as metagenomics, transcriptomics, and metabolomics, to conduct multidimensional systemic mechanism analysis. The focus will be on multi-omics synergistic strategies to elucidate the dynamic evolutionary patterns of metabolic features and their triggering mechanisms across different stages of composting in microbial communities. By dissecting the contributions of specific microbial groups and their dynamic responses to the humification process, efficient regulatory strategies can be developed to shorten the composting humification cycle and enhance the application value of the resulting products. This research will simultaneously provide intuitive and refined scientific evidence for the association between microbial metabolic activities and the humification process, ultimately establishing robust theoretical foundations and technological pathways for the sustainable management of organic waste.

## References

[1] Zhang Z, Yang H, Wang B, Chen C, Zou X, Cheng T, Li J. 2023. Aerobic co-composting of mature compost with cattle manure: organic matter conversion and microbial community characterization. Bioresour Technol 382:129187. doi:10.1016/j.biortech.2023.129187 37196747

[2] Xu Z, Gao X, Li G, Nghiem LD, Luo W, Zhang F. 2024. Microbial sources and sinks of nitrous oxide during organic waste composting. Environ Sci Technol 58:7367–7379. doi:10.1021/acs.est.3c10341 38644786

[3] Chen L, Yi Z, Chen Y, Li Y, Jiang H, Wang J, Zhao M, Chen Y, Zhang W, Chi N, Zeng G. 2025. Electro-Fenton-assisted Camellia oleifera by-product composting for reduction of greenhouse gas emission and improvement of humification. Chem Eng J 504:158901. doi:10.1016/j.cej.2024.158901

[4] Jiang H, Yi Z, Chen Y, Li Y, Zhao M, Wang J, Liu Y, Jia X, Luo M, Nie Y, Chi N. 2025. Innovative application of recyclable magnetic LDO beads for enhancing the remediation of Cd and Pb contamination and the humification during composting. J Hazard Mater 495:138863. doi:10.1016/j.jhazmat.2025.138863 40505397

[5] Chen Y, Chen Y, Li Y, Wu Y, Zeng Z, Xu R, Wang S, Li H, Zhang J. 2019. Changes of heavy metal fractions during co-composting of agricultural waste and river sediment with inoculation of Phanerochaete chrysosporium. J Hazard Mater 378:120757. doi:10.1016/j.jhazmat.2019.120757 31207488

[6] Bui VKH, Truong HB, Hong S, Li X, Hur J. 2023. Biotic and abiotic catalysts for enhanced humification in composting: a comprehensive review. J Clean Prod 402:136832. doi:10.1016/j.jclepro.2023.136832

[7] Li S, Sun K, Latif A, Si Y, Gao Y, Huang Q. 2022. Insights into the applications of extracellular laccase-aided humification in livestock manure composting. Environ Sci Technol 56:7412–7425. doi:10.1021/acs.est.1c08042 35638921

[8] Chen L, Yi Z, Chen Y, Li Y, Jiang H, Wang J, Chen Y, Nie Y, Luo M, Wang Q, Zhang W, Wu Y. 2024. Improved humification and Cr(VI) immobilization by CaO2 and Fe3O4 during composting. Bioresour Technol 413:131479. doi:10.1016/j.biortech.2024.131479 39265754

[9] Zhang Y, Yang B, Peng S, Zhang Z, Cai S, Yu J, Wang D, Zhang W. 2024. Mechanistic insights into chemical conditioning on transformation of dissolved organic matter and plant biostimulants production during sludge aerobic composting. Water Res 255:121446. doi:10.1016/j.watres.2024.121446 38489963

[10] Kong Y, Zhang J, Zhang X, Gao X, Yin J, Wang G, Li J, Li G, Cui Z, Yuan J. 2024. Applicability and limitation of compost maturity evaluation indicators: a review. Chem Eng J 489:151386. doi:10.1016/j.cej.2024.151386

[11] Zheng Y, Feng Z, Wang P, Xu S, Gao X, Ren L, Yang T, Zhao X, Wei Y, Li J. 2023. Suppressive performance of food waste composting with polylactic acid: emphasis on microbial core metabolism pathways and mechanism. Bioresour Technol 384:129339. doi:10.1016/j.biortech.2023.129339 37343797

[12] Xu P, Shu L, Li Y, Zhou S, Zhang G, Wu Y, Yang Z. 2023. Pretreatment and composting technology of agricultural organic waste for sustainable agricultural development. Heliyon 9:e16311. doi:10.1016/j.heliyon.2023.e16311 37305492 PMC10256924

[13] Chen L, Chen Y, Li Y, Liu Y, Jiang H, Li H, Yuan Y, Chen Y, Zou B. 2023. Improving the humification by additives during composting: a review. Waste Manag 158:93–106. doi:10.1016/j.wasman.2022.12.040 36641825

[14] Xing R, Yin K, Du X, Lin Y, Yu Z, Chen Z, Zhou S. 2024. Enhanced organic matter humification by hydroxyl radical generation during electric field-assisted aerobic composting. Chem Eng J 482:148910. doi:10.1016/j.cej.2024.148910

[15] Zhu L, Zhao Y, Chen S, Miao X, Fang Z, Yao X, Dong C, Hu B. 2024. Alternating ventilation accelerates the mineralization and humification of food waste by optimizing the temperature-oxygen-moisture distribution in the static composting reactor. Bioresour Technol 393:130050. doi:10.1016/j.biortech.2023.130050 37989420

[16] Mo J, Zhao C, Fang C, Yu W, Long Y, Mei Q, Wu W. 2025. Pre-biodrying treatment enhances lignin-related pathways with phenolic hydroxyls as reactive cores to accelerate humification during composting. Bioresour Technol 416:131786. doi:10.1016/j.biortech.2024.131786 39522621

[17] Chen L, Chen Y, Li Y, Jiang H, Liu Y, Yuan Y, Wang J, Li H, Chen Y. 2024. Passivation of heavy metals during co-composting of Camellia oleifera by-products and river sediment with the additives of MnO2-loaded biochar. Ind Crops Prod 215:118697. doi:10.1016/j.indcrop.2024.118697

[18] Zhao Y, Liu Z, Zhang B, Cai J, Yao X, Zhang M, Deng Y, Hu B. 2023. Inter-bacterial mutualism promoted by public goods in a system characterized by deterministic temperature variation. Nat Commun 14:5394. doi:10.1038/s41467-023-41224-7 37669961 PMC10480208

[19] He Y, Chen W, Xiang Y, Zhang Y, Xie L. 2024. Unveiling the effect of PFOA presence on the composting process: roles of oxidation stress, carbon metabolism, and humification process. J Hazard Mater 479:135682. doi:10.1016/j.jhazmat.2024.135682 39236542

[20] Wei Z, Ahmed Mohamed T, Zhao L, Zhu Z, Zhao Y, Wu J. 2022. Microhabitat drive microbial anabolism to promote carbon sequestration during composting. Bioresour Technol 346:126577. doi:10.1016/j.biortech.2021.126577 34923079

[21] Xie T, Zhang Z, Yu Y, Tian Y, Wang F, Li D, Nan J, Feng Y. 2023. Aeration intensity drives dissolved organic matter transformation and humification during composting by regulating the organics metabolic functions of microbiome. Chem Eng J 476:146645. doi:10.1016/j.cej.2023.146645

[22] Wu D, Xia T, Zhang Y, Wei Z, Qu F, Zheng G, Song C, Zhao Y, Kang K, Yang H. 2021. Identifying driving factors of humic acid formation during rice straw composting based on Fenton pretreatment with bacterial inoculation. Bioresour Technol 337:125403. doi:10.1016/j.biortech.2021.125403 34147772

[23] Wen X, Sun R, Cao Z, Huang Y, Li J, Zhou Y, Fu M, Ma L, Zhu P, Li Q. 2022. Synergistic metabolism of carbon and nitrogen: cyanate drives nitrogen cycle to conserve nitrogen in composting system. Bioresour Technol 361:127708. doi:10.1016/j.biortech.2022.127708 35907603

[24] Wang Y, Li J, Chang Y, Chang S, Chen Y, Wei D, Li R, Zheng Y, Kang Z, Wu Z, Chen P, Wei Y, Li J, Xu Z. 2024. Metabolomics analysis of advancing humification mechanism in secondary fermentation of composting by fungal bioaugmentation. Science of The Total Environment 933:173267. doi:10.1016/j.scitotenv.2024.173267 38754504

[25] Xu Z, Gao X, Li G, Nghiem LD, Luo W. 2023. Microbes from mature compost to promote bacterial chemotactic motility via tricarboxylic acid cycle-regulated biochemical metabolisms for enhanced composting performance. Bioresour Technol 387:129633. doi:10.1016/j.biortech.2023.129633 37544546

[26] Wu J, Yao W, Zhao L, Zhao Y, Qi H, Zhang R, Song C, Wei Z. 2022. Estimating the synergistic formation of humus by abiotic and biotic pathways during composting. J Clean Prod 363:132470. doi:10.1016/j.jclepro.2022.132470

[27] Wang S, Wang Y, He X, Lu Q. 2022. Degradation or humification: rethinking strategies to attenuate organic pollutants. Trends Biotechnol 40:1061–1072. doi:10.1016/j.tibtech.2022.02.007 35339288

[28] Cai S, Liu M, Zhang Y, Hu A, Zhang W, Wang D. 2022. Molecular transformation of dissolved organic matter and formation pathway of humic substances in dredged sludge under aerobic composting. Bioresour Technol 364:128141. doi:10.1016/j.biortech.2022.128141 36257519

[29] Cai D, Lu Y, Zhu Y, Wang D, Shi J, Liu L, Li J, Zhan X, Zhang W, Xu H. 2025. Inducing hour-level humification of Enteromorpha prolifera to fabricate fulvic-like acid fertilizer with Fenton’s reagent. Nat Commun 16:5860. doi:10.1038/s41467-025-61204-3 40595651 PMC12217275

[30] Chen J, Sun T, Yang P, Peng S, Yu J, Wang D, Zhang W. 2024. Inhibitory effect of microplastics derived organic matters on humification reaction of organics in sewage sludge under alkali-hydrothermal treatment. Water Res 252:121231. doi:10.1016/j.watres.2024.121231 38324988

[31] Cai S, Zhang W, Yang B, Zhang Y, Sun P, Cai Z, Xiang L, Wang D, Zhang W. 2024. Alkali-thermal humification treatment for simultaneous plant-growth-promoting compounds production and antibiotic removal from lincomycin fermentation residues. Chem Eng J 485:149449. doi:10.1016/j.cej.2024.149449

[32] Zhou J, Gao W, Xie L, Zhang R, Zhang Y, Wei Z. 2024. Revealing mechanism of phenol-amine reaction to form humus in compost based on high-resolution liquid chromatography mass spectrometry and spectroscopy. Bioresour Technol 403:130862. doi:10.1016/j.biortech.2024.130862 38768664

[33] Cai S, Zhang Y, Hu A, Liu M, Wu H, Wang D, Zhang W. 2023. Dissolved organic matter transformation mechanisms and process optimization of wastewater sludge hydrothermal humification treatment for producing plant biostimulants. Water Res 235:119910. doi:10.1016/j.watres.2023.119910 37001233

[34] Zhao P, Du Z, Fu Q, Ai J, Hu A, Wang D, Zhang W. 2023. Molecular composition and chemodiversity of dissolved organic matter in wastewater sludge via Fourier transform ion cyclotron resonance mass spectrometry: Effects of extraction methods and electrospray ionization modes. Water Res 232:119687. doi:10.1016/j.watres.2023.119687 36758353

[35] Zhu Y, Cao Y, Fu B, Wang C, Shu S, Zhu P, Wang D, Xu H, Zhong N, Cai D. 2024. Waste milk humification product can be used as a slow release nano-fertilizer. Nat Commun 15:128. doi:10.1038/s41467-023-44422-5 38167856 PMC10761720

[36] Sun R, Cao Z, Wen X, Ma L, Zhou Y, Li J, Fu M, Zhu P, Li K, Li Q. 2023. Quinone redox cycling drives lignocellulose depolymerization and degradation in composting environments based on metagenomics analysis. Sci Total Environ 856:159009. doi:10.1016/j.scitotenv.2022.159009 36162579

[37] Marzban N, Libra JA, Rotter VS, Herrmann C, Ro KS, Filonenko S, Hoffmann T, Antonietti M. 2024. Maximizing the value of liquid products and minimizing carbon loss in hydrothermal processing of biomass: an evolution from carbonization to humification. Biochar 6:44. doi:10.1007/s42773-024-00334-1

[38] Xu Z, Li R, Zhang X, Wang S, Xu X, Ho Daniel Tang K, Emmanuel Scriber K II, Zhang Z, Quan F. 2024. Molecular mechanisms of humus formation mediated by new ammonifying microorganisms in compost. Chem Eng J 483:149341. doi:10.1016/j.cej.2024.149341

[39] Wang X, Tian L, Li Y, Zhong C, Tian C. 2022. Effects of exogenous cellulose-degrading bacteria on humus formation and bacterial community stability during composting. Bioresour Technol 359:127458. doi:10.1016/j.biortech.2022.127458 35700902

[40] Lin B, Zhang Y, Hao Y, Lu M, Xiang H, Ding D, Niu S, Li K, Li J, Huang Z. 2025. Insights into nitrogen metabolism and humification process in aerobic composting facilitated by microbial inoculation. Environ Res 269:120894. doi:10.1016/j.envres.2025.120894 39828197

[41] Manzoni S. 2017. Flexible carbon-use efficiency across litter types and during decomposition partly compensates nutrient imbalances-results from analytical stoichiometric models. Front Microbiol 8:661. doi:10.3389/fmicb.2017.00661 28491054 PMC5405148

[42] Jiang H, Yi Z, Chen Y, Li Y, Wang J, Chen L, He Y, Wang N, Wang Q, Chen Y, Zhang W. 2025. Exogenous laccase drives synergistic immobilization of heavy metals in agriculture waste and soil composting: deciphering straw degradation-humification-microbial interactions. Chem Eng J 515:163595. doi:10.1016/j.cej.2025.163595

[43] Mo J, Fang C, Qin Y, Zhao C, Mei Q, Wu W. 2024. Ammonia assimilation coupled with rapid humification increases recalcitrant nitrogen reservoirs during bioaugmented mechanical composting. J Clean Prod 447:141628. doi:10.1016/j.jclepro.2024.141628

[44] Dong H, Zeng Q, Sheng Y, Chen C, Yu G, Kappler A. 2023. Coupled iron cycling and organic matter transformation across redox interfaces. Nat Rev Earth Environ 4:659–673. doi:10.1038/s43017-023-00470-5

[45] Guo H, Zhao Y, Chang J-S, Lee D-J. 2023. Enzymes and enzymatic mechanisms in enzymatic degradation of lignocellulosic biomass: a mini-review. Bioresour Technol 367:128252. doi:10.1016/j.biortech.2022.128252 36334864

[46] Yao X, Liu Q, Li D. 2024. Mechanism underlying effects of cellulose-degrading microbial inoculation on amino acid degradation and biosynthesis during composting. Bioresour Technol 403:130899. doi:10.1016/j.biortech.2024.130899 38801951

[47] Wu D, Yue J, Gao W, Wang F, Qu F, Song C, Wei Z. 2024. Functional genes are the key factors driving the Fenton-like reactions to promote the hydrolysis of lignocellulosic biomass during composting. Ind Crops Prod 210:118131. doi:10.1016/j.indcrop.2024.118131

[48] Sun Z, Chu L, Wang X, Fang G, Liu C, Chen H, Gu C, Gao J. 2023. Roles of natural phenolic compounds in polycyclic aromatic hydrocarbons abiotic attenuation at soil–air interfaces through oxidative coupling reactions. Environ Sci Technol 57:11967–11976. doi:10.1021/acs.est.3c02032 37478127

[49] Wu D, Ren H, Zhao Y, Wei Z, Li J, Song C. 2023. Effect of Fenton-like reactions on the hydrolysis efficiency of lignocellulose during rice straw composting based on genomics and metabolomics sequencing. J Clean Prod 396:136493. doi:10.1016/j.jclepro.2023.136493

[50] Su Q, Wu Y, Wang S, Li Y, Zhao J, Huang F, Wu J. 2023. The reverse function of lignin-degrading enzymes: the polymerization ability to promote the formation of humic substances in domesticated composting. Bioresour Technol 380:129059. doi:10.1016/j.biortech.2023.129059 37075849

[51] Li H, Liu C, Ni J-Q, Zhuo G, Li Y, Zheng Y, Zhen G. 2025. Impact of cellulolytic nitrogen-fixing composite inoculants on humification pathways and nitrogen cycling in kitchen waste composting. Bioresour Technol 416:131819. doi:10.1016/j.biortech.2024.131819 39547296

[52] Wu D, Wei Z, Mohamed TA, Zheng G, Qu F, Wang F, Zhao Y, Song C. 2022. Lignocellulose biomass bioconversion during composting: mechanism of action of lignocellulase, pretreatment methods and future perspectives. Chemosphere 286:131635. doi:10.1016/j.chemosphere.2021.131635 34346339

[53] Liu Y, Tang Y, Gao H, Zhang W, Jiang Y, Xin F, Jiang M. 2021. Challenges and future perspectives of promising biotechnologies for lignocellulosic biorefinery. Molecules 26:5411. doi:10.3390/molecules26175411 34500844 PMC8433869

[54] Qiao C, Ryan Penton C, Liu C, Shen Z, Ou Y, Liu Z, Xu X, Li R, Shen Q. 2019. Key extracellular enzymes triggered high-efficiency composting associated with bacterial community succession. Bioresour Technol 288:121576. doi:10.1016/j.biortech.2019.121576 31176934

[55] Niu Q, Meng Q, Yang H, Wang Y, Li X, Li G, Li Q. 2021. Humification process and mechanisms investigated by Fenton-like reaction and laccase functional expression during composting. Bioresour Technol 341:125906. doi:10.1016/j.biortech.2021.125906 34523564

[56] Simmons TJ, Frandsen KEH, Ciano L, Tryfona T, Lenfant N, Poulsen JC, Wilson LFL, Tandrup T, Tovborg M, Schnorr K, Johansen KS, Henrissat B, Walton PH, Lo Leggio L, Dupree P. 2017. Structural and electronic determinants of lytic polysaccharide monooxygenase reactivity on polysaccharide substrates. Nat Commun 8:1064. doi:10.1038/s41467-017-01247-3 29057953 PMC5651836

[57] Li X, Li S, Liang X, McClements DJ, Liu X, Liu F. 2020. Applications of oxidases in modification of food molecules and colloidal systems: laccase, peroxidase and tyrosinase. Trends in Food Science & Technology 103:78–93. doi:10.1016/j.tifs.2020.06.014

[58] Ye J, Hu A, Gao C, Li F, Li L, Guo Y, Ren G, Li B, Rensing C, Nealson KH, Zhou S, Xiong Y. 2024. Abiotic methane production driven by ubiquitous non‐fenton‐type reactive oxygen species. Angew Chem Int Ed 63. doi:10.1002/anie.202403884 38489233

[59] Sun H, Xing R, Ye X, Yin K, Zhang Y, Chen Z, Zhou S. 2023. Reactive oxygen species accelerate humification process during iron mineral-amended sludge composting. Bioresour Technol 370:128544. doi:10.1016/j.biortech.2022.128544 36584721

[60] Jiang Z, Li X, Li M, Zhu Q, Li G, Ma C, Li Q, Meng J, Liu Y, Li Q. 2021. Impacts of red mud on lignin depolymerization and humic substance formation mediated by laccase-producing bacterial community during composting. J Hazard Mater 410:124557. doi:10.1016/j.jhazmat.2020.124557 33234392

[61] Liang C, Schimel JP, Jastrow JD. 2017. The importance of anabolism in microbial control over soil carbon storage. Nat Microbiol 2:17105. doi:10.1038/nmicrobiol.2017.105 28741607

[62] Yang H, Ma L, Fu M, Li K, Li Y, Li Q. 2023. Mechanism analysis of humification coupling metabolic pathways based on cow dung composting with ionic liquids. J Environ Manage 325:116426. doi:10.1016/j.jenvman.2022.116426 36240639

[63] Liu Q, He X, Wang K, Li D. 2023. Biochar drives humus formation during composting by regulating the specialized metabolic features of microbiome. Chem Eng J 458:141380. doi:10.1016/j.cej.2023.141380

[64] Zhu L, Liu L, Tan C, Li C, Le B, Yao X, Hu B. 2025. Sustainable decentralized food waste composting using a pulse alternating ventilation pilot-scale device: case study based on LCA and LCC analysis. Bioresour Technol 419:132078. doi:10.1016/j.biortech.2025.132078 39814154

[65] He Y, Zhang Y, Huang X, Xu J, Zhang H, Dai X, Xie L. 2022. Deciphering the internal driving mechanism of microbial community for carbon conversion and nitrogen fixation during food waste composting with multifunctional microbial inoculation. Bioresour Technol 360:127623. doi:10.1016/j.biortech.2022.127623 35850391

[66] Kong W, Sun B, Zhang J, Zhang Y, Gu L, Bao L, Liu S. 2020. Metagenomic analysis revealed the succession of microbiota and metabolic function in corncob composting for preparation of cultivation medium for Pleurotus ostreatus. Bioresour Technol 306:123156. doi:10.1016/j.biortech.2020.123156 32179397

[67] Liu Q, He X, Luo G, Wang K, Li D. 2022. Deciphering the dominant components and functions of bacterial communities for lignocellulose degradation at the composting thermophilic phase. Bioresour Technol 348:126808. doi:10.1016/j.biortech.2022.126808 35131458

[68] Duan M, Zhang Y, Zhou B, Qin Z, Wu J, Wang Q, Yin Y. 2020. Effects of Bacillus subtilis on carbon components and microbial functional metabolism during cow manure-straw composting. Bioresour Technol 303:122868. doi:10.1016/j.biortech.2020.122868 32032936

[69] Yu C, Lu Q, Fu C, Jiang Z, Huang J, Jiang F, Wei Z. 2022. Exploring the internal driving mechanism underlying bacterial community-induced organic component conversion and humus formation during rice straw composting with tricarboxylic acid cycle regulator addition. Bioresour Technol 365:128149. doi:10.1016/j.biortech.2022.128149 36265785

[70] Tang Y, Khan E, Tsang DCW. 2024. Waste nitrogen upcycling to amino acids during anaerobic fermentation on biochar: an active strategy for regulating metabolic reducing power. Environ Sci Technol 58:20060–20072. doi:10.1021/acs.est.4c08890 39485020

[71] Tang Y, Xie H, Sun J, Li X, Zhang Y, Dai X. 2022. Alkaline thermal hydrolysis of sewage sludge to produce high-quality liquid fertilizer rich in nitrogen-containing plant-growth-promoting nutrients and biostimulants. Water Res 211:118036. doi:10.1016/j.watres.2021.118036 35032873

[72] Klier KM, Anantharaman K. 2026. An updated view of metabolic handoffs in microbiomes. Trends Microbiol 34:98–112. doi:10.1016/j.tim.2025.07.009 40885659

[73] Yu Z, Liu X, Zhao M, Zhao W, Liu J, Tang J, Liao H, Chen Z, Zhou S. 2019. Hyperthermophilic composting accelerates the humification process of sewage sludge: molecular characterization of dissolved organic matter using EEM–PARAFAC and two-dimensional correlation spectroscopy. Bioresour Technol 274:198–206. doi:10.1016/j.biortech.2018.11.084 30504103

[74] Chen S, Hua Y, Song Q, Yuan X, Yang J, Zhang Y, Dai X. 2025. Sewage sludge valorization via phytohormones production: parameter regulation and process evaluation. Water Res 270:122813. doi:10.1016/j.watres.2024.122813 39580943

[75] Lin C, Xin Z, Yuan S, Sun J, Dong B, Xu Z. 2024. Effects of production temperature on the molecular composition and seed-germination-promoting properties of sludge-based hydrochar-derived dissolved organic matter. Water Res 251:121133. doi:10.1016/j.watres.2024.121133 38237463

[76] Mo J, Xin L, Zhao C, Qin Y, Nan Q, Mei Q, Wu W. 2023. Reducing nitrogen loss during kitchen waste composting using a bioaugmented mechanical process with low pH and enhanced ammonia assimilation. Bioresour Technol 372:128664. doi:10.1016/j.biortech.2023.128664 36702327

[77] Liu R, Xu Y, Cao J, Geng H, Chen R, Liu H, Chen Y, Yuan S, Dai X. 2024. Effects of pH-varying thermal modification on sewage sludge: a focus on releasing nitrogen- and phosphorus-containing substances. Water Res 257:121746. doi:10.1016/j.watres.2024.121746 38733966

[78] Wang K, Mao H, Wang Z, Tian Y. 2018. Succession of organics metabolic function of bacterial community in swine manure composting. J Hazard Mater 360:471–480. doi:10.1016/j.jhazmat.2018.08.032 30144766

[79] Jia Y, Wang P, Ou Y, Yan Y, Zhou S, Sun L, Lu H. 2022. Insights into the microbial response mechanisms to ciprofloxacin during sulfur-mediated biological wastewater treatment using a metagenomics approach. Water Res 223:118995. doi:10.1016/j.watres.2022.118995 36007398

[80] Kamimura N, Takahashi K, Mori K, Araki T, Fujita M, Higuchi Y, Masai E. 2017. Bacterial catabolism of lignin-derived aromatics: new findings in a recent decade: Update on bacterial lignin catabolism. Environ Microbiol Rep 9:679–705. doi:10.1111/1758-2229.12597 29052962

[81] Wu Y, Zhu X, Wang X, Lin Z, Reinfelder JR, Li F, Liu T. 2023. A new electron shuttling pathway mediated by lipophilic phenoxazine via the interaction with periplasmic and inner membrane proteins of Shewanella oneidensis MR-1. Environ Sci Technol 57:2636–2646. doi:10.1021/acs.est.2c07862 36652548

[82] Zhang X, Liu Y, Zhou Q, Bai Y, Li R, Li T, Li J, Alessi DS, Konhauser KO. 2023. Exogenous electroactive microbes regulate soil geochemical properties and microbial communities by enhancing the reduction and transformation of Fe(III) minerals. Environ Sci Technol 57:7743–7752. doi:10.1021/acs.est.3c00407 37171176

[83] Liu X, Li P, Bao K, Wang Y, Wang H, Wang Y, Jiang Z, Yang Y, Yuan S, Kappler A, Wang Y. 2025. Synergistic interaction between microbial nitrogen fixation and iron reduction in the environment. ISME J 19:wraf212. doi:10.1093/ismejo/wraf212 40981677 PMC12516963

[84] Xing R, Sun H, Du X, Lin H, Qin S, Chen Z, Zhou S. 2023. Enhanced degradation of microplastics during sludge composting via microbially-driven Fenton reaction. J Hazard Mater 449:131031. doi:10.1016/j.jhazmat.2023.131031 36821904

[85] Han R, Lv J, Huang Z, Zhang S, Zhang S. 2020. Pathway for the production of hydroxyl radicals during the microbially mediated redox transformation of iron (Oxyhydr)oxides. Environ Sci Technol 54:902–910. doi:10.1021/acs.est.9b06220 31886656

[86] Gu C, Wang J, Guo M, Sui M, Lu H, Liu G. 2018. Extracellular degradation of tetrabromobisphenol A via biogenic reactive oxygen species by a marine Pseudoalteromonas sp. Water Res 142:354–362. doi:10.1016/j.watres.2018.06.012 29908463

[87] Zhang X, Guo Y, Liu G, Liu Y, Shi J, Hu L, Zhao L, Li Y, Yin Y, Cai Y, Jiang G. 2023. Superoxide-mediated extracellular mercury reduction by aerobic marine bacterium Alteromonas sp. KD01. Environ Sci Technol 57:20595–20604. doi:10.1021/acs.est.3c04777 38007712

[88] Xing R, Yang X, Sun H, Ye X, Liao H, Qin S, Chen Z, Zhou S. 2022. Extensive production and evolution of free radicals during composting. Bioresour Technol 359:127491. doi:10.1016/j.biortech.2022.127491 35724905

[89] Chen Y, Chen Y, Li Y, Liu Y, Li H, Jiang H, Luo X, Tang P, Chen L, Yan H. 2021. Evolution of humic substances and the forms of heavy metals during co-composting of rice straw and sediment with the aid of Fenton-like process. Bioresour Technol 333:125170. doi:10.1016/j.biortech.2021.125170 33932807

[90] Zhao X, Dang Q, Zhang C, Yang T, Gong T, Xi B. 2023. Revisiting organic waste-source-dependent molecular-weight governing the characterization within humic acids liking to humic-reducing microorganisms in composting process. J Hazard Mater 442:130049. doi:10.1016/j.jhazmat.2022.130049 36179623

[91] Li M, Jiang H, Li R, Liu W, Xie Y, Wu W, Liu D, Wu M, Qiu Z. 2025. Effects of biochar-loaded microbial agent in regulating nitrogen transformation and integration into humification for straw composting. Bioresour Technol 417:131873. doi:10.1016/j.biortech.2024.131873 39586479

[92] Zhang W, Zhao Y, Lu Q, Feng W, Wang L, Wei Z. 2022. Evaluating differences in humic substances formation based on the shikimic acid pathway during different materials composting. Bioresour Technol 364:128060. doi:10.1016/j.biortech.2022.128060 36195217

[93] Wen X, Zhou Y, Liang X, Li J, Huang Y, Li Q. 2023. A novel carbon-nitrogen coupled metabolic pathway promotes the recyclability of nitrogen in composting habitats. Bioresour Technol 381:129134. doi:10.1016/j.biortech.2023.129134 37164230

[94] Liang X, Wen X, Yang H, Lu H, Wang A, Liu S, Li Q. 2024. Incorporating microbial inoculants to reduce nitrogen loss during sludge composting by suppressing denitrification and promoting ammonia assimilation. Sci Total Environ 915:170000. doi:10.1016/j.scitotenv.2024.170000 38242453

[95] Wei Z, Zhao Y, Zhao L, Wang L, Wu J. 2023. The contribution of microbial shikimic acid to humus formation during organic wastes composting: a review. World J Microbiol Biotechnol 39:240. doi:10.1007/s11274-023-03674-5 37392253

[96] Tang Y, Dong B, Dai X. 2022. Hyperthermophilic pretreatment composting to produce high quality sludge compost with superior humification degree and nitrogen retention. Chem Eng J 429:132247. doi:10.1016/j.cej.2021.132247

